# Direct benefit transfer for nutritional support of patients with TB in India—analysis of national TB program data of 3.7 million patients, 2018–2022

**DOI:** 10.1186/s12889-024-17777-7

**Published:** 2024-01-25

**Authors:** Kathiresan Jeyashree, Prema Shanmugasundaram, Devika Shanmugasundaram, Sri Lakshmi Priya G, Jeromie W V Thangaraj, Sumitha TS, Sumit Pandey, Sabarinathan Ramasamy, Rahul Sharma, Sivavallinathan Arunachalam, Vaibhav Shah, Venkateshprabhu Janagaraj, Sivakami Sundari S, Joshua Chadwick, Hemant Deepak Shewade, Aniket Chowdhury, Swati Iyer, Raghuram Rao, Sanjay K Mattoo, Manoj V Murhekar

**Affiliations:** 1 ICMR-National Institute of Epidemiology, R-127, TNHB, Ayapakkam, Chennai, Tamil Nadu 600077 India; 2 TB support network, WHO Country Office for India, New Delhi, India; 3 Central TB Division, New Delhi, India

**Keywords:** *Ni-kshay Poshan Yojana*, Direct benefit transfer, Nutritional support, National TB Program, Undernutrition, India

## Abstract

**Background:**

Patients with TB have additional nutritional requirements and thus additional costs to the household. *Ni-kshay Poshan Yojana*(NPY) is a Direct Benefit Transfer (DBT) scheme under the National Tuberculosis Elimination Programme(NTEP) in India which offers INR 500 monthly to all notified patients with TB for nutritional support during the period of anti-TB treatment. Five years after its implementation, we conducted the first nationwide evaluation of NPY.

**Methods:**

In our retrospective cohort study using programmatic data of patients notified with TB in nine randomly selected Indian states between 2018 and 2022, we estimated the proportion of patients who received at least one NPY instalment and the median time to receive the first instalment. We determined the factors associated (i) with non-receipt of NPY using a generalised linear model with Poisson family and log link and (ii) with time taken to receive first NPY benefit in 2022 using quantile regression at 50th percentile.

**Results:**

Overall, 3,712,551 patients were notified between 2018 and 2022. During this period, the proportion who received at least one NPY instalment had increased from 56.9% to 76.1%. Non-receipt was significantly higher among patients notified by private sector (aRR 2.10;2.08,2.12), reactive for HIV (aRR 1.69;1.64,1.74) and with missing/undetermined diabetic status (aRR 2.02;1.98,2.05). The median(IQR) time to receive the first instalment had reduced from 200(109,331) days in 2018 to 91(51,149) days in 2022. Patients from private sector(106.9;106.3,107.4days), those with HIV-reactive (103.7;101.8,105.7days), DRTB(104.6;102.6,106.7days) and missing/undetermined diabetic status (115.3;114,116.6days) experienced longer delays.

**Conclusions:**

The coverage of NPY among patients with TB had increased and the time to receipt of benefit had halved in the past five years. Three-fourths of the patients received at least one NPY instalment, more than half of whom had waited over three months to receive the first instalment. NTEP has to focus on timely transfer of benefits to enable patients to meet their additional nutritional demands, experience treatment success and avoid catastrophic expenditure.

**Supplementary Information:**

The online version contains supplementary material available at 10.1186/s12889-024-17777-7.

## Background

Poverty, food insecurity, undernutrition, overcrowding and other forms of deprivation have been well established as proximate risk factors of tuberculosis (TB), in addition to being implicated in the reduced access to TB care, high TB incidence and mortality [1–3]. Undernutrition is a leading population-level risk factor for tuberculosis (TB) in India [4], with almost half of the active TB cases attributable to undernutrition [5]. India’s national TB prevalence survey reported Body Mass Index (BMI) < 18.5 as a critical risk factor for TB [6]. Undernutrition can compromise the immunity of patients with TB, affect the pharmacodynamics and pharmacokinetics and tolerance to anti-tuberculosis therapy (ATT) and increase the risk of unfavourable TB treatment outcomes [7]. There are additional nutritional requirements for the patients with TB and these requirements translate into additional costs for an affected household [8, 9]. Such costs hinder the attainment of the End TB strategy goal of achieving zero catastrophic costs due to TB.

Social protection interventions, including poverty eradication schemes for patients with TB and TB-affected households, are increasingly being incorporated into national-level TB prevention and control strategies [10]. Various strategies, including cash transfers [11–13], additional rations for TB-affected households, and ready-to-use therapeutic foods for patients and household contacts, have been tested and found to have varying levels of effectiveness and feasibility [14–18]. Nutritional support in kind constituted the highest proportion (30%) of schemes in LMIC for TB-affected households, followed by conditional cash transfer schemes [19]. Nutritional support increases treatment success, reduces loss to follow-up [20], decreases death during treatment among patients with TB and also reduces the incidence of the disease among household contacts of a patient with TB [18, 21]. These strategies have been designed to target all patients with TB or specific populations like older persons, household contacts of patients with TB or patients with TB and comorbidities and have been led by the state or Non-Government Organizations.

The National Tuberculosis Elimination Programme (NTEP) in India offers various Direct Benefit Transfer (DBT) schemes like the *Ni-kshay Poshan Yojana (*NPY; *Ni-kshay*- End TB, *Poshan-* Nutrition, *Yojana*- Scheme) for nutrition of patients, transport support for patients with TB in notified tribal areas, honorarium for treatment supporters, and notification and treatment outcome incentive for private sector providers and incentives for Accredited Social Health Activist (ASHA) workers for seeding patient bank account within 15 days. The NPY is a TB-specific cash transfer scheme initiated on April 1, 2018 which credits INR 500 (about US$ 7) [22] monthly to the bank account of patients with TB who are notified under the NTEP through Public Financial Management System (PFMS). This benefit is provided throughout the duration of their anti- TB treatment towards meeting their additional nutritional requirements [23].

Earlier studies on NPY implementation had reported low coverage during the early stages of implementation [24–27], and had identified several process related challenges such as lack of bank account [24, 25, 27], multiple steps of the credit process [27], over burdening of staff [24, 25], and inadequate support from private practitioners [26]. However, the evidence is from studies conducted in smaller geographical confines on limited number of patients with a shorter duration of follow-up. Five years since its implementation, a nationwide evaluation of the NPY is due to provide policy relevant insights about the program performance. Therefore, this study aims to analyse nationally representative data to determine the proportion of patients who received at least one NPY instalment and the time interval between TB diagnosis and receipt of the first NPY instalment.

## Methods

### Study setting

India is administratively divided into 28 States and 8 Union Territories which are further divided into districts. India is one of the high TB burden countries, with 2,422,121 cases notified in 2022 of whom, around 30.3% were notified from the private sector [28]. Under the NTEP, the district TB centres monitor the programme implementation in each district through a network of Tuberculosis Units and Peripheral Health Institutions. The State TB cell governs all district TB centres under it. The NTEP data is entered, analysed, visualised and reported using *Ni-kshay* which is a web enabled patient management system.

All the states and Union Territories of India implement NPY. All patients notified with TB are eligible to receive NPY, INR 500 per month, for the entire duration of their anti-TB treatment. In the event of prolongation of treatment duration due to regimen change or failure of treatment, the monthly benefit will be credited for the additional period as well. The first two NPY instalments are credited together as INR1000 (US$14) soon after diagnosis, followed by a monthly instalment of INR500 from the third month of treatment.

On diagnosis, the bank details of the beneficiary are collected, validated by the program officials in district and state levels before forwarding to the PFMS. On approval of the bank account details by the PFMS, the benefit will be credited. Every month, a list of benefits to be credited to patients in a given district is generated, validated and approved followed by credit of instalments. So, a patient will be included in every month’s beneficiary list until his/her TB treatment outcome is declared. If there is an error at any level, the details will have to be checked and revalidated for approval from the district level [23]. Since 2021, all the Indian states have adopted a Single Nodal Agency (SNA), under which the funds of all Government schemes, including NPY, will be released [29, 30].

### Study design and population

We conducted a retrospective cohort study using programmatic data of all patients with TB, notified between 2018 and 2022 extracted from *Ni-kshay*. All patients notified in a given year were considered as the notification cohort for that year. We used standard operational definitions recommended in the WHO definitions and reporting framework [31].

### Sampling of study sites

We divided the states of India into three strata (high, medium and low) based on TB score, which is a composite score measuring program performance at sub-national level (Table S1), where higher scores meant better program performance. We classified TB score > 80 as high, 60 to 80 as medium and < 60 as low [32]. We selected three states from each stratum by simple random sampling. Delhi, Rajasthan and Bihar were selected under low TB score, Uttarakhand, Telangana and Tamil Nadu under medium TB score and Meghalaya, Odisha and Gujarat were selected from high TB score strata. All the patients with TB notified under NTEP in the selected states were included in the study.

### Data collection and analysis

We extracted the NTEP data for the years 2018–2022 from *Ni-kshay* registers and reports. We obtained patients’ demographic and clinical characteristics, and treatment outcome from current notification register, number of instalments and amount credited per instalment from NPY beneficiary register and the dates of credit of each instalment from DBT Turn-around time (TAT) indicator register. We appended and merged the data from the three registers based on the unique episode ID and analysed using Stata v.17.0 and R v.4.3.1.

We calculated the proportion of patients who had received at least one NPY instalment and median(IQR) time interval (in days) between date of diagnosis and date of credit of the first NPY instalment stratum level. We used the survey data analysis module of Stata v.17.0 to estimate the proportion of receipt of NPY instalment along with 95% Confidence Interval (CI) at the country level after adjusting for stratification and clustering. We have also calculated the proportion who received at least INR 3000 as NPY benefit, stratified by treatment outcomes.

Our dataset was censored on July 7, 2023. Thus, patients notified in the last two quarters of 2022 had a follow-up period ranging from 6 months to 12 months compared to the patients notified earlier who had a follow-up of more than 12 months. Hence, we present the indicators for 2022 as an overall estimate for the year and quarter-wise to understand the possible impact of this shorter follow-up time on the indicators.

We determined the factors associated with non-receipt of first NPY instalment in 2022 using a generalised linear model with Poisson family and log link and calculated the adjusted relative risk(aRR) with 95% CI. Since the sample size was large, even if statistical significance is achieved, we have considered an aRR cut off of < 0.7 and > 1.3, and a difference in marginal means of at least 10 days as programmatically relevant and significant [33].

We calculated the median time interval (measured in terms of days) between date of diagnosis and date of credit of the first NPY instalment in various sub-groups of interest. We determined the factors associated with time taken to receive at least one NPY benefit in 2022 using quantile regression at 50th percentile and presented marginal means along with 95% CI.

## Results

### Sociodemographic and clinical characteristics

Between 2018 and 2022, a total of 3,712,551 patients with TB were notified from the nine states. Among them, 2,349,504 (63.3%) were male, 2,862,414 (77.1%) were between 15 and 59 years and 1,001,829 (27%) were notified by the private sector. About three quarters (*n* = 2,726,671, 76.8%) had pulmonary TB, 102,285 (2.8%) patients had drug resistant TB (DRTB), 65,771 (1.8%) were HIV reactive and 217,879 (5.9%) had diabetes. Unfavourable treatment outcome was reported in 583,173 (16.1%) patients (Table 1).

**Table 1:** Socio demographic and clinical characteristics of patients who received atleast one *Ni-kshay Poshan Yojana* instalment, India, 2018–2022 (*N* = 3,712,551)

Variable	Category	Total	Receipt of at least one NPY instalment n (%)			
		N (%)	Overall	Low TB Score	Medium TB Score	High TB Score
	Overall	3,712,551	2,640,069 (71.1)	1,176,728 (64.4)	694,746 (77.4)	768,595 (77.9)
Notified Year	2018	683,074 (18.4)	388,905 (56.9)	165,619 (51.6)	105,786 (62.9)	117,500 (60.6)
	2019	813,643 (21.9)	546,266 (67.1)	225,631 (57.5)	151,362 (73.0)	169,273 (79.3)
	2020	637,793 (17.2)	481,074 (75.4)	212,583 (67.6)	125,706 (81.1)	142,785 (84.9)
	2021	740,734 (20.0)	586,451 (79.2)	269,190 (72.2)	144,649 (85.0)	172,612 (87.3)
	2022	837,307 (22.6)	637,373 (76.1)	303,705 (71.2)	167,243 (84.8)	166,425 (78.0)
Age (in Years)	Below 15	228,045 (6.1)	152,307 (66.8)	91,104 (62.7)	25,992 (74.0)	35,211 (73.8)
	15–59	2,862,414 (77.1)	2,045,350 (71.5)	904,169 (64.7)	529,759 (77.7)	611,422 (78.1)
	60 and above	622,035 (16.8)	442,377 (71.1)	181,438 (63.7)	138,980 (76.9)	121,959 (78.1)
Gender	Male	2,349,504 (63.3)	1,660,109 (70.7)	718,025 (63.7)	446,299 (76.7)	495,785 (77.3)
	Female	1,359,926 (36.6)	978,225 (71.9)	457,884 (65.5)	247,966 (78.5)	272,375 (79.0)
	Transgender	2284 (0.1)	1460 (64.0)	632 (55.3)	443 (73.0)	385 (72.1)
Notifying Sector	Public	2,710,721 (73.0)	2,124,808 (78.4)	892,200 (70.9)	584,100 (84.3)	648,508 (85.4)
	Private	1,001,829 (27.0)	515,260 (51.4)	284,528 (49.9)	110,646 (54.0)	120,086 (52.9)
Type of case	New	3,177,594 (85.6)	2,236,712 (70.4)	1,007,893 (63.7)	598,429 (76.8)	630,390 (77.1)
	PMDT	102,285 (2.8)	80,762 (79.0)	37,888 (73.8)	23,173 (84.5)	19,701 (83.9)
	Retreatment	432,672 (11.7)	322,595 (74.6)	130,947 (67.1)	73,144 (79.8)	118,504 (81.3)
Site of Disease	Extra Pulmonary	825,605 (23.2)	628,239 (76.1)	282,075 (70.8)	160,961 (80.4)	185,203 (81.7)
	Pulmonary	2,726,671 (76.8)	2,001,515 (73.4)	888,933 (67.9)	531,550 (78.6)	581,032 (78.4)
Drug Type	DSTB	3,610,266 (97.2)	2,559,307 (70.9)	1,138,840 (64.1)	671,573 (77.1)	748,894 (77.8)
	DRTB	102,285 (2.8)	80,762 (79.0)	37,888 (73.8)	23,173 (84.5)	19,701 (83.9)
HIV status	Non-Reactive	3,089,052 (83.2)	2,382,703 (77.1)	1,035,429 (72.2)	620,906 (81.6)	726,368 (81.3)
	Reactive	65,771 (1.8)	44,479 (67.6)	11,165 (53.9)	19,620 (76.5)	13,694 (70.5)
	Missing/Undetermined	557,728 (15.0)	212,887 (38.2)	130,134 (34.9)	54,220 (48.7)	28,533 (38.9)
Diabetic	Yes	217,879 (5.9)	184,561 (84.7)	45,395 (73.6)	95,574 (89.5)	43,592 (88.2)
	No	2,602,310 (70.1)	2,044,964 (78.6)	901,356 (74.1)	470,913 (83.3)	672,695 (82.0)
	Missing/Undetermined	892,362 (24.0)	410,544 (46.0)	229,977 (41.8)	128,259 (56.8)	52,308 (44.8)

Abbreviations: PMDT, Programmatic Management of Drug Resistant Tuberculosis; HIV, Human Immunodeficiency Virus; DRTB, Drug resistant Tuberculosis; DSTB, Drug sensitive Tuberculosis

### Receipt of at least one NPY instalment

**Fig. 1:**
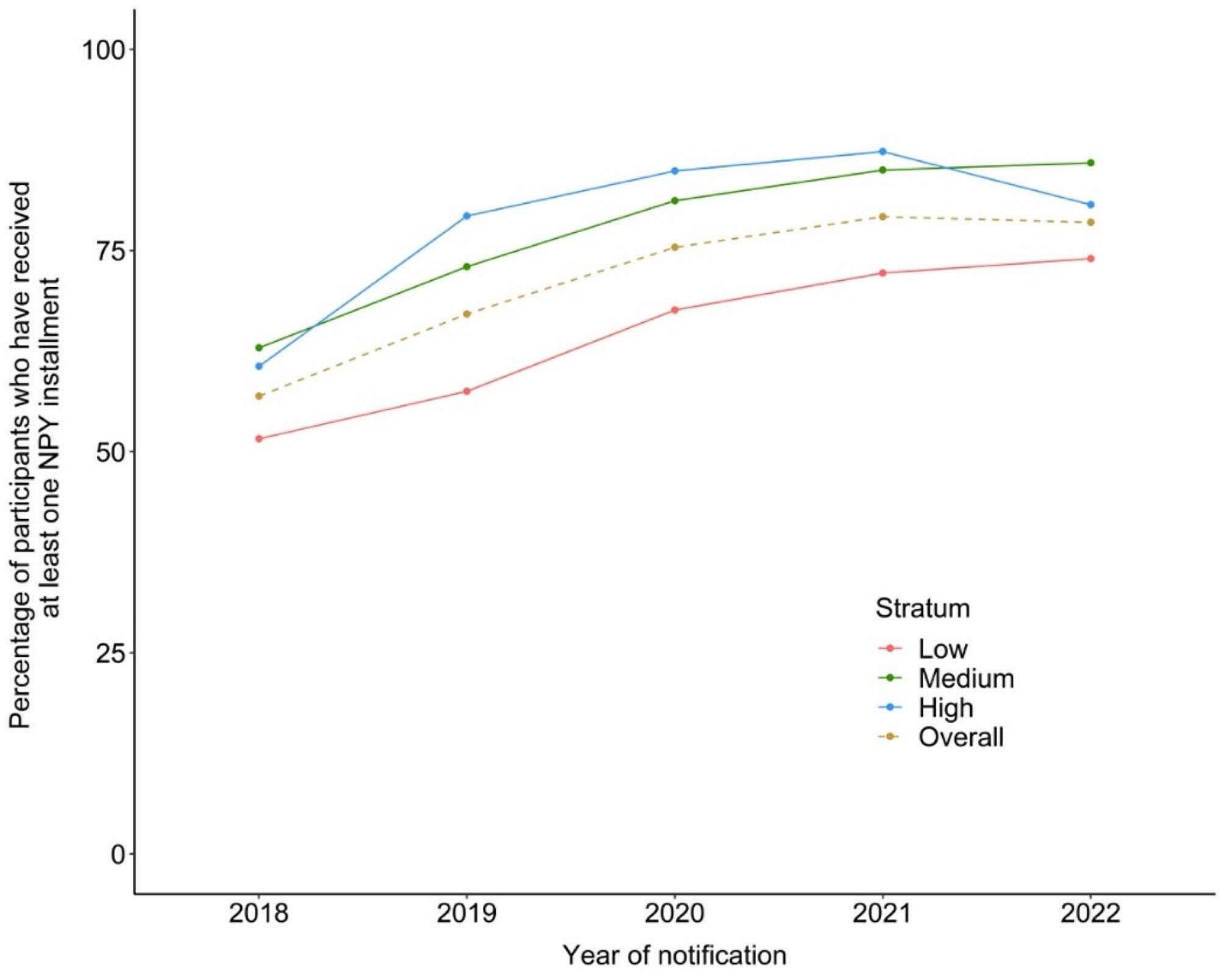
Stratum-wise and overall proportion of patients who received atleast one *Ni-kshay Poshan Yojana* instalment, India, 2018–2022

**Fig. 2:**
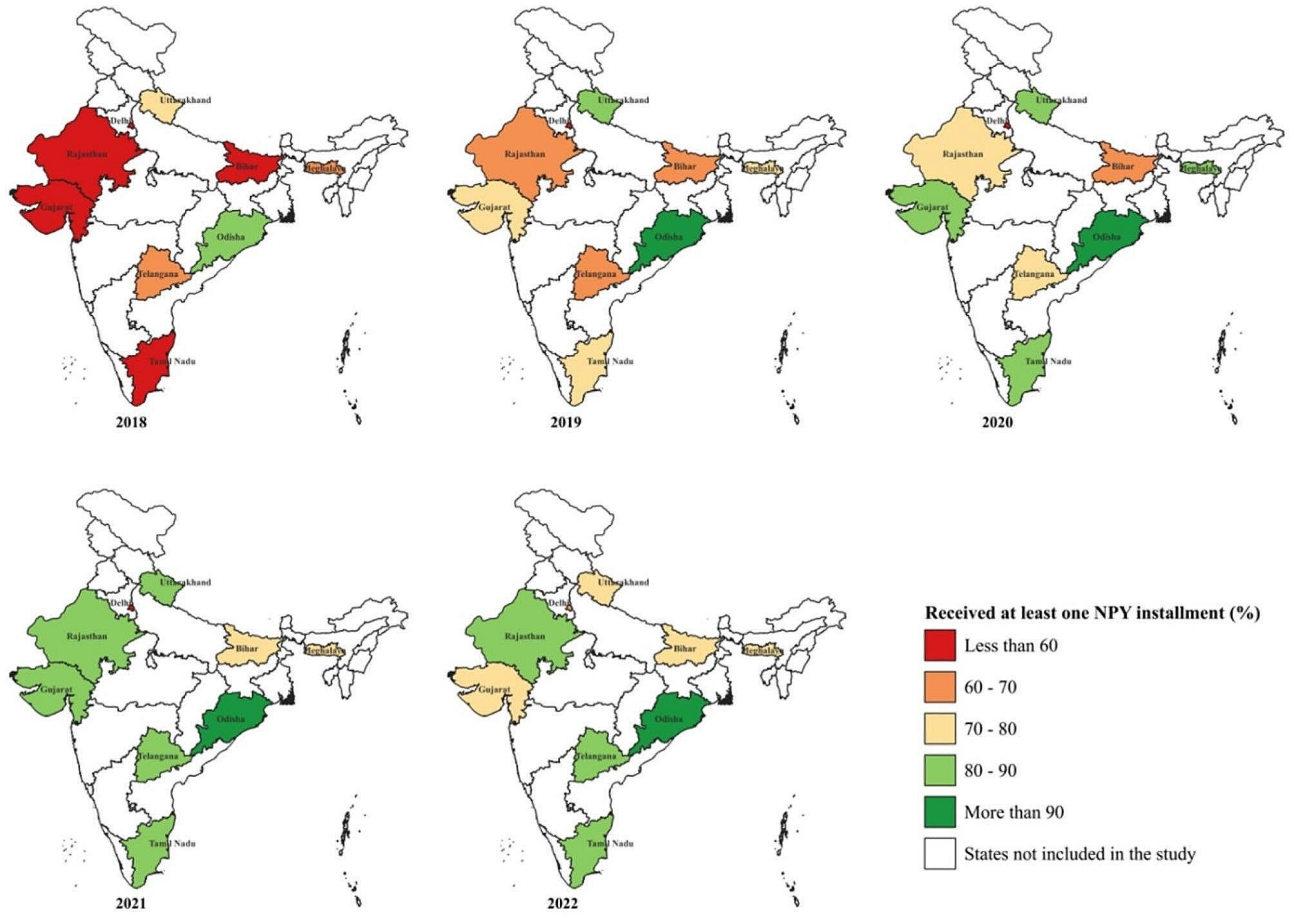
Proportion of patients with TB receiving at least one *Ni-kshay Poshan Yojana* instalment in India, 2018–2022

Overall, 2,640,069 (71.1%; 95% CI 65.8, 75.9) patients had received at least one instalment of NPY. This proportion increased from 56.9% in 2018 to 76.1% in 2022 (Table 1; Fig. 1). The proportion of patients who had received at least one NPY instalment was the lowest in the low TB score stratum (64.4%) followed by the medium (77.4%) and high (77.9%) strata (Table 1). Among those who received at least one instalment, the proportion of patients who received a benefit amount INR 3000 or more was 64.6% in 2018, 76.8% in 2021 and 67.5% in 2022 (Table 2). Within strata, the proportion of patients who had received the first NPY instalment was highest in Odisha (91.7%) and least in Delhi (51.8%) (Fig. 2, Table S2). The percentage improvement in NPY receipt in 2022 since 2018 was highest in Delhi (67.2%), followed by Tamil Nadu (45.3%) and Gujarat (45.2%). States that had reported high NPY receipt in 2018 itself like Odisha (85.1%) and Uttarakhand (72.9%) registered a 10% increase in performance in 2022.

**Fig. 3:**
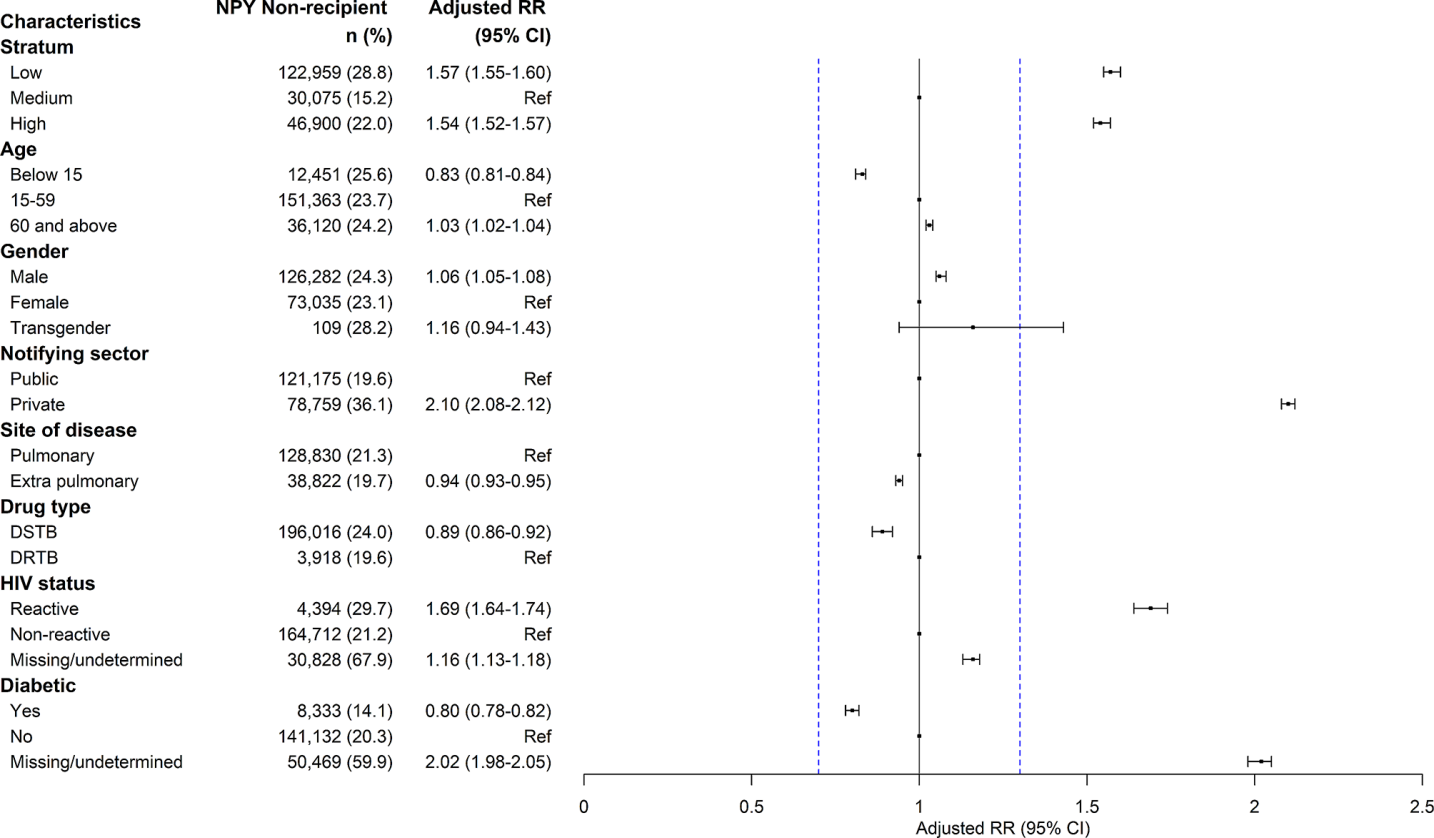
Factors associated with non-receipt of *Ni-kshay Poshan Yojana* among patients with TB notified in India, 2022 (*N* = 837,307) *Note*: Since the sample size is large, we have considered an RR cut off of < 0.7 and > 1.3 (indicated in blue line) as programmatically significant DRTB- Drug resistant Tuberculosis; DSTB- Drug sensitive Tuberculosis; HIV- Human Immunodeficiency Virus

In 2022, patients from low TB score stratum (aRR 1.57; 95%CI 1.55, 1.60), high TB score stratum (aRR 1.54; 1.52, 1.57), notified by the private sector (aRR 2.10; 95%CI 2.08,2.12), reactive for HIV (aRR 1.69; 95%CI 1.64,1.74) and with missing/undetermined diabetic status (aRR 2.02; 95%CI 1.98,2.05) were less likely to have received any NPY benefit compared to their counterparts (Fig. 3).

Of the 104,623 (12.5%) who suffered unfavorable TB treatment outcomes in 2022, 48,903 (46.7%) had received the benefit of whom 31,864 (65.1%) received it after their treatment outcome was declared (Figure S1).

**Table 2:** Treatment outcome of patients with TB who received the *Ni-kshay Poshan Yojana*, India, 2018–2022 (*n* = 2,640,069)

Notified Year	Overall		Treatment Outcome^a^			
			Unfavourable		Favourable	
	≥ INR 3000	< INR 3000	≥ INR 3000	< INR 3000	≥ INR 3000	< INR 3000
2018	251,125 (64.6)	137,780 (35.4)	7774 (24.5)	23,945 (75.5)	243,333 (68.1)	113,821 (31.9)
2019	427,702 (78.3)	118,564 (21.7)	13,413 (24.8)	40,757 (75.2)	414,210 (84.2)	77,723 (15.8)
2020	382,944 (79.6)	98,130 (20.4)	11,892 (24.1)	37,516 (75.9)	370,853 (86.0)	60,355 (14.0)
2021	450,248 (76.8)	136,203 (23.2)	12,157 (22.2)	42,684 (77.8)	434,995 (82.5)	92,518 (17.5)
2022	430,380 (67.5)	206,993 (32.5)	8435 (17.2)	40,468 (82.8)	398,211 (75.0)	132,952 (25.0)
2022 Q1	131,325 (82.3)	28,323 (17.7)	3055 (23.6)	9872 (76.4)	126,078 (87.5)	18,035 (12.5)
2022 Q2	146,832 (81.1)	34,164 (18.9)	2939 (20.7)	11,276 (79.3)	140,177 (86.7)	21,566 (13.3)
2022 Q3	110,874 (69.7)	48,142 (30.3)	1881 (15.7)	10,122 (84.3)	101,462 (75.5)	32,947 (24.5)
2022 Q4	41,349 (30.0)	96,364 (70.0)	560 (5.7)	9198 (94.3)	30,494 (33.5)	60,404 (66.5)

^a^Treatment outcome yet to be declared for 62,057 patients; INR 3000 (US$36.4) refers to the total amount that a person with TB will receive after 6 months of anti-TB treatment

### Time to receive the first NPY instalment

**Fig. 4:**
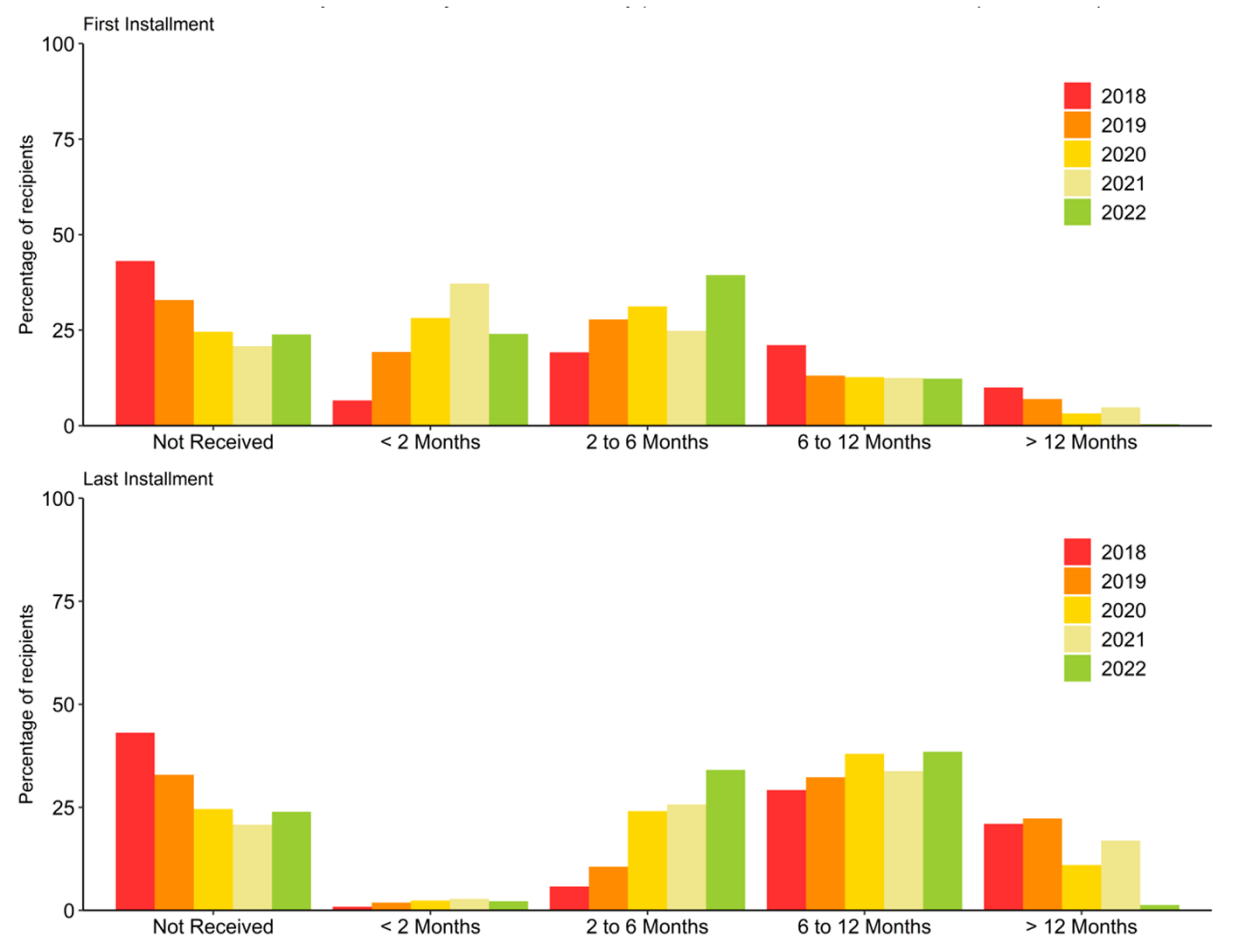
Time to receive *Ni-kshay Poshan Yojana* instalments by patients with TB, India, 2018–2022 (*N* = 3,712,551) *Note*: ^*^The first ever instalment of NPY received by patient since diagnosis ^#^ The last instalment of NPY credited to the patient

Overall, the median (IQR) time interval between diagnosis and receipt of first NPY instalment was 96 (48, 193) days. It had reduced from 200 (109,331) days in 2018 to 91 (51,149) days in 2022. The proportion who had received their first instalment within 6 months of diagnosis had increased from 30.3% in 2018 to 67.7% in 2022 (Fig. 4, Figure S2). Among the nine states, the patients in Odisha had the shortest time to receipt of the first NPY instalment with a median (IQR) of 63 (35,122) days, followed by Tamil Nadu with 68 (38,123) days. Patients in Delhi experienced the longest time to receipt of NPY 136 (75,246) days (Table S3).

In 2022, the marginal mean (95% CI) time to receipt of first NPY instalment of patients in high TB score stratum were significantly longer (108.4; 107.9, 108.9) days, compared to low TB score stratum (98.4; 98.0, 98.7) days. Patients notified from private sector (106.9; 106.3, 107.4) days, those who were HIV reactive (103.7; 101.8, 105.7) days, suffered from DRTB (104.6; 102.6, 106.7) days and those with undetermined diabetic status (115.3; 114, 116.6) days experienced significantly longer time to receipt of first NPY instalment compared to their counterparts (Table 3, Table S4).

## Discussion

We report the extent of implementation of NPY in India between 2018 and 2022 in a nationally representative sample of 3.7 million patients with TB notified under the NTEP. Nearly three-fourths of the patients had received at least one NPY instalment in 2022. Despite an increase in coverage and significant reduction in delay in credit of benefits, patients with TB continue to experience a median delay of three months to receive their first NPY instalment.

There has been a significant increase in the proportion receiving at least one NPY benefit between 2018 and 2022 in all three strata. This is also corroborative with the estimates from earlier, smaller scale evaluations of NPY from India reporting coverage less than 50%. With efforts geared towards ending TB by 2025, NPY implementation has benefited from the higher levels of political commitment. The Government of India through its Jan Dhan Yojana facilitated opening of bank accounts for people with zero bank balance also [34] which has majorly removed possession of a bank account as a barrier to receiving NPY [24–26, 35, 36]. Digitalisation of health information systems and banking services has contributed to more efficient and error free processing under NPY.

The progress has not been uniform across the states. In our study, patients from low TB score stratum were more likely to not receive the benefit. The TB score measures the overall performance of the NTEP in a given state and it is reasonable to expect that it will also reflect on the performance of the state in timely disbursal of NPY which is also implemented through the NTEP program structure and staff. There could have been specific state-led or district-led initiatives [37] in addition to those under NTEP that could have also have contributed to such differential performance in NPY credit [38]. The performance of states also depends on its socio-economic, health and health system indicators [39].

The time to receipt of NPY benefit had reduced by half between 2018 and 2022. However, patients still face long delays in receiving their benefits. Notably, two thirds of the patients with unfavorable treatment outcomes in our study received their first benefit after the outcome was declared. This is critical because the ultimate vision of NPY is to reduce unfavorable treatment outcomes by enabling patients to access better nutrition, thus improving their immunity, tolerance of ATT and promoting higher adherence to ATT [9] and treatment success [40]. It is possible that these unfavorable outcomes could have been prevented by timely disbursal of benefits. The delay can also push them into financial catastrophe. Similar or longer delays have been reported in India [24–26].

Delay in NPY receipt can be due to challenges that the patients face in opening or sharing bank account details, inadequate information about the purpose of and procedures involved in NPY or lack of motivation to avail the benefit which they may find as insufficient [24]. There could also be provider and system related delays in creating the beneficiary lists, verifying their credentials and approving the benefit at PFMS. Rejections due to change in beneficiary details or bank account details, or merger of banks are other reasons for delay for individual patients or in some cases for whole batches of benefits from a given district or state [24]. Though mostly digitalised, these manual links in the process of approval of beneficiary list every month at the district office and PFMS delay the transfer of benefits [26]. Insufficient funds at the state level could be another reason for delay in crediting benefits even after approval. The delay in 2022 might also have been because of the acclimatization to the implementation of Single Nodal Agency (SNA) [30]. Due to the differential pace in rolling out SNA, states in medium and high TB score strata experienced longer delay in 2022 compared to the low TB score states which are yet to implement SNA fully. This delay is likely to reduce significantly after smoothening out the initial hiccups in SNA implementation.

Private sector notified patients were significantly less likely to receive benefit and also experience longer delays. This could be due to patients’ unwillingness to provide their details because of social stigma [41], wealthy patients who forego the benefits [24], difficulty faced by NTEP staff or private provider support agencies (PPSAs) in following up the private sector notified patients and lack of support from private providers [26, 41]. Similarly, non-receipt and longer delays were common among HIV reactive patients. This could be due to the single window system of delivery of services wherein patients receive antiretroviral therapy and ATT through the National AIDS Control program [42]. This might cause these patients to be left out from the routine follow-up interactions with the NTEP staff causing delays in obtaining details for their registering for NPY and thus leading to non-receipt and longer delays. Patients with undetermined/missing diabetic status probably represent the patient sub-group who are left out from other services offered under NTEP like assessment of diabetic status and linkage with the non-communicable diseases program of the district. These patients with undetermined comorbidity status indicate the need for better functioning of NTEP in terms of addressing co-morbidities that increase risk of unfavourable TB treatment outcomes and TB transmission and the need to foster effective linkages with the respective health programs.

### Strengths and limitations

Our study has several strengths. Ours is the first country wide evaluation of the NPY since its inception and the largest of the evaluations of benefit transfer schemes for patients with TB worldwide. Our analysis includes data of over 3.7 million patients over the past five years from a nationally representative sample of states at different level of programmatic performance in the country. While most available studies have studied cash transfer schemes in controlled trial settings [16], our analysis captures the real world scenario of the scheme delivered within a health program. We use available program data to provide policy relevant insights for optimal implementation of the NPY scheme. The variables on the generation of NPY benefits, status of credit, and dates of credit, are auto-generated by the system leaving no room for errors due to manual entry or bias in analysis. We provide a patient wise analysis of receipt and time to receipt instead of a benefit wise analysis, enabling realistic interpretations relevant to patients and program managers.

Our study is not without limitations. Analysis of program data meant that there would be missing data. However, the missing data on our outcome variables and key explanatory variables were less than 5% and are less likely to have influenced our results. Late in 2021, the option to forego NPY benefits was offered to patients and captured as a variable in *Ni-kshay*. For the sake of comparability across years, we have included these patients as non-recipients. However, due to minuscule numbers opting for it (data not shown), we believe that this hasn’t influenced our estimates of coverage. The high median delay of over three months to receive the first NPY instalment precluded any attempt to look for association between receipt of NPY benefit and TB treatment outcomes, given that unfavourable outcomes are more likely in the first two months of beginning ATT [43, 44]. Our analysis is restricted only to the variables available in the *Ni-kshay* portal. The non-uniform and non-standardised recording of data on patient’s nutritional status also limited us from looking for impact on NPY benefit receipt on nutritional status. There are no variables in *Ni-kshay* that measure the socioeconomic status of the patient and household.

## Conclusion

The coverage of NPY benefit among patients with TB has increased significantly since 2018 and almost three-fourths of the patients notified with TB in 2022 had received benefits. The time to receipt of NPY benefit to the patient has reduced over time but is still very high. There is scope for expanding the coverage to more patients with TB and ensuring timely payments to the patients to promote better TB treatment outcomes.

### Recommendations

In the light of our findings, we make the following recommendations.

1. Timely credit of first payment must be ensured by identifying and addressing system and beneficiary side delays, to enable patients to optimally utilise the benefit to meet their nutritional needs.
2. The program needs to sensitise and actively engage the private sector in NPY through PPSAs and the public private mix coordinator employed under NTEP, to facilitate timely transfer of the benefit to patients notified from private sector. Strengthening the linkage between NTEP and National Acquired Immunodeficiency syndrome (AIDS) control Program can improve the NPY benefit receipt.
3. There is a need to standardise the measurement and recording of height and weight of patients with TB at diagnosis, at every follow up sputum testing visit and at treatment outcome assignment to enable analysis of term impact of NPY benefit on nutritional status.
4. Given the complex causal pathway between receipt of a cash benefit for nutritional support and TB treatment outcomes, future operational research may focus on novel designs and modelling methods to elicit this impact addressing confounders and mediators.

**Table 3:** Factors associated with time to receipt of first *Ni-kshay Poshan Yojana* instalment in patients, India, 2022 (*N* = 837,307)

Variable	Category	Total N (%)	Recipients of NPY n (%)	Time to receipt (Days) Median (IQR)	Adjusted Coefficient 95% CI	Marginal means (Days) 95% CI	p value
	Overall	837,307	637,373 (76.1)	91 (51, 149)			
Stratum	Low	42,664 (50.9)	303,705 (71.2)	98 (59, 153)	-10 (-10.60, -9.40)	98.38 (98.03, 98.74)	< 0.001
	Medium	197,318 (23.6)	167,243 (84.8)	66 (37, 112)	-41 (-41.67, -40.33)	67.38 (66.91, 67.86)	< 0.001
	High	213,325 (25.5)	166,425 (78.0)	101 (58, 177)	Reference	108.38 (107.91, 108.86)	
Age (in Years)	Below 15	48,571 (5.8)	36,120 (74.4)	102 (58, 163)	5 (3.92, 6.08)	98.24 (97.20, 99.28)	< 0.001
	15–59	639,394 (76.4)	488,031 (76.3)	91 (51, 150)	Reference	93.24 (92.96, 93.52)	
	60 and above	149,341 (17.8)	113,221 (75.8)	84 (47, 138)	-4 (-4.64, -3.36)	89.24 (88.66, 89.82)	< 0.001
Gender	Female	315,931 (37.7)	242,896 (76.9)	93 (53, 154)	Reference	94.66 (94.27, 95.06)	
	Male	520,222 (62.1)	393,940 (75.7)	89 (50, 145)	-3 (-3.51, -2.49)	91.66 (91.36, 91.97)	< 0.001
	Transgender	387 (0.1)	278 (71.8)	91 (56, 132)	-3 (-14.68, 8.68)	91.66 (79.99, 103.34)	0.62
Notifying Sector	Public	619,220 (74.0)	498,045 (80.4)	87 (49, 142)	Reference	88.87 (88.60, 89.14)	
	Private	218,087 (26.0)	139,328 (63.9)	104 (58, 169)	18 (17.40, 18.60)	106.87 (106.34, 107.40)	< 0.001
Site of Disease	Extra Pulmonary	197,317 (23.6)	158,495 (80.3)	92 (53, 151)	0 (-0.58, 0.58)	92.80 (92.30, 93.30)	1.00
	Pulmonary	604,704 (72.2)	475,874 (78.7)	90 (50, 148)	Reference	92.80 (92.52, 93.08)	
Drug Type	DSTB	817,327 (97.6)	621,311 (76.0)	90 (51, 148)	-12 (-14.07, -9.93)	92.63 (92.39, 92.87)	< 0.001
	DRTB	19,980 (2.4)	16,062 (80.4)	101 (60, 161)	Reference	104.63 (102.58, 106.68)	
HIV status	Non-Reactive	777,120 (92.8)	612,408 (78.8)	90 (51, 148)	Reference	92.73 (92.48, 92.98)	
	Reactive	14,771 (1.8)	10,377 (70.3)	92 (51, 155)	11 (9.06, 12.94)	103.73 (101.81, 105.65)	< 0.001
	Missing/Undetermined	45,416 (5.4)	14,588 (32.1)	106 (62, 167)	-5 (-7.00, -3.00)	87.73 (85.75, 89.70)	< 0.001
Diabetic	Yes	59,051 (7.0)	50,718 (85.9)	74 (40, 120)	-7 (-7.91, -6.09)	85.29 (84.42, 86.16)	< 0.001
	No	693,992 (82.9)	552,860 (79.7)	91 (51, 149)	Reference	92.29 (92.03, 92.55)	
	Missing/Undetermined	84,264 (10.1)	33,795 (40.1)	113 (67, 178)	23 (21.65, 24.35)	115.29 (113.99, 116.60)	< 0.001
Treatment Outcome	Unfavourable	104,623 (12.5)	48,903 (46.7)	91 (50, 153)	0 (-0.91, 0.91)	92.80 (91.93, 93.67)	1.00
	Favourable	643,253 (76.8)	531,163 (82.6)	91 (51, 152)	Reference	92.80 (92.55, 93.05)	

Abbreviations: HIV, Human Immunodeficiency Virus; DRTB, Drug resistant Tuberculosis; DSTB, Drug sensitive Tuberculosis

### Electronic supplementary material

Below is the link to the electronic supplementary material.

Supplementary Material 1

## Data Availability

The datasets used and analysed during the current study are available from the corresponding author on reasonable request.

## Abbreviations

ASHA: Accredited Social Health Activist
ATT: Anti-Tuberculosis Therapy
BMI: Body Mass Index
CI: Confidence Interval
CSS: Centrally Sponsored Schemes
DBT TAT: Direct Benefit Transfer Turn Around Time
DBT: Direct Benefit Transfer
DRTB: Drug Resistant Tuberculosis
DTC: District TB Centres
GoI: Government of India
HIV: Human Immunodeficiency Virus
IHEC: Institutional Human Ethics Committee
IQR: Inter Quartile Range
LMIC: Low- or Middle-Income Country
NPY: *Ni-kshay Poshan Yojana*
NTEP: National TB Elimination Programme
PFMS: Public Financial Management System
PHI: Peripheral Health Institutions
RR: Relative risk
SNA: Single Nodal Agency
TB: Tuberculosis
TU: Tuberculosis Units
