# Supplementary material for: Direct benefit transfer for nutritional support of patients with TB in India—analysis of national TB program data of 3.7 million patients, 2018–2022
Source: BMC Public Health. 2024 Jan 25;24:299. doi: 10.1186/s12889-024-17777-7 (PMC10811802; doi:10.1186/s12889-024-17777-7)
Supplement: Supplementary file 1 — Supplementary Material 1 [file 12889_2024_17777_MOESM1_ESM.pdf]

Additional file 1

Table S1 Indicators used to calculate TB score of the states

S.No	Parameter	Indicator	Score allotted
1	Achievement of TB notification among the target identified	% of Target TB notification achieved	20
2	HIV testing/ screening of TB notified patients	% of net TB notified patients with known HIV status	10
3	UDST coverage among the TB notified patients	% of net TB notified patients with UDST done	10
4	Treatment Success Rate of TB notified patients	Treatment Success Rate for net TB patients	15
5	*Ni-kshay Poshan Yojana* implementation	% of Eligible beneficiaries paid at least once under *Ni-kshay Poshan Yojana*	10
6	DRTB treatment initiation among the diagnosed patients	% of MDR/ RR patients initiated on treatment out of net diagnosed	15
7	Utilisation of the allotted budget	% of expenditure amongst ROP	10
8	Latent TB infection management	% of children <5/<6 years given chemoprophylaxis against the total eligible children identified	5
		% of PLHIV given IPT against total eligible PLHIV	5

TB – Tuberculosis; HIV – Human Immunodeficiency Virus; UDST – Universal Drug Susceptibility Testing; MDR - Multi-drug resistant; RR - Rifampicin Resistant; ROP - Record of Proceedings; PLHIV - People Living with HIV; IPT - Isoniazid Preventive Therapy

Source: India Tuberculosis report 2022, Central TB Division, Ministry of Health and Family Welfare, Government of India

Table S2 State wise receipt of at least one *Ni-Kshay Poshan Yojana* instalment among patients with TB in India, 2018-2022 (N =3712551)

Stratum	States	2018<br>n (%)	2019<br>n (%)	2020<br>n (%)	2021<br>n (%)	2022<br>n (%)	Overall<br>n (%)
Low	Bihar	50849 (50.6)	76186 (60.7)	67825 (66.2)	98806 (72.4)	112409 (67.4)	406075 (64.3)
	Delhi	27112 (37.4)	42689 (44.6)	42173 (55.6)	48211 (56.7)	55999 (63.3)	216184 (51.8)
	Rajasthan	87658 (59.3)	106756 (62.3)	102585 (75.1)	122173 (80.8)	135297 (79)	554469 (71.2)
Medium	Tamil Nadu	57705 (59.4)	83821 (74.9)	62408 (86.9)	74812 (87.6)	83720 (86.8)	362466 (78.4)
	Telangana	33301 (65.4)	47147 (66.8)	45716 (72.8)	50594 (82.9)	62292 (84.9)	239050 (75.0)
	Uttarakhand	14780 (72.9)	20394 (81.9)	17582 (87.0)	19243 (80.9)	21231 (77.2)	93230 (79.9)
High	Gujarat	73444 (52.1)	116637 (75.0)	96838 (81.7)	120036 (85.0)	106910 (72.2)	513865 (72.9)
	Meghalaya	2935 (65.9)	3850 (73.0)	3339 (80.8)	3026 (71.9)	3817 (76.2)	16967 (73.5)
	Odisha	41121 (85.1)	48786 (92.3)	42608 (93.6)	49550 (94.7)	55698 (92.5)	237763 (91.7)
Overall		388905 (56.9)	546266 (67.1)	481074 (75.4)	586451 (79.2)	637373 (76.1)	2640069 (71.1)

Table S3 State wise time to receipt of first *Ni-Kshay Poshan Yojana* instalment among patients with TB in India, 2018-2022 (N =3712551)

Stratum	States	2018	2019	2020	2021	2022	Overall
		Median (IQR) days	Median (IQR) days	Median (IQR) days	Median (IQR) days	Median (IQR) days	Median (IQR) days
Low	Bihar	202 (115, 342)	105 (57, 210)	91 (48, 185)	144 (67, 304)	123 (70, 188)	125 (65, 230)
	Delhi	208 (125, 347)	161 (91, 281)	90 (52, 159)	195 (77, 360)	119 (73, 177)	136 (75, 246)
	Rajasthan	237 (153, 357)	195 (91, 387)	174 (105, 261)	100 (54, 187)	80 (50, 115)	132 (70, 240)
Medium	Tamil Nadu	191 (112, 300)	78 (44, 137)	55 (35, 91)	48 (31, 76)	56 (34, 94)	68 (38, 123)
	Telangana	132 (58, 257)	89 (29, 203)	110 (66, 197)	60 (36, 111)	61 (36, 103)	79 (41, 155)
	Uttarakhand	168 (79, 304)	71 (33, 152)	43 (27, 76)	68 (31, 217)	190 (119, 261)	95 (40, 212)
High	Gujarat	188 (77, 365)	90 (48, 168)	60 (35, 100)	34 (20, 62)	139 (81, 209)	79 (37, 170)
	Meghalaya	215 (137, 348)	113 (64, 201)	66 (35, 119)	281 (70, 380)	114 (72, 166)	126 (64, 244)
	Odisha	194 (107, 323)	81 (47, 135)	45 (29, 75)	37 (24, 60)	59 (37, 94)	63 (35, 122)
Overall		200 (109, 331)	105 (54, 213)	82 (43, 161)	65 (34, 157)	91 (51, 149)	96 (48, 193)

IQR – Interquantile range

Table S4 Stratum wise time to receipt of first *Ni-Kshay Poshan Yojana* instalment among patients with TB in India, 2018-2022 (N =3712551)

Variable	Category	Total N (%)	Receipt of at least one NPY instalment n (%)	Time to receipt of first NPY instalment (in days) Median (IQR)			
				Overall	Low TB Score	Medium TB Score	High TB Score
Overall		3712551	2640069 (71.1)	96 (48, 193)	130 (69, 238)	74 (39, 145)	74 (36, 157)
Notified year	2018	683074 (18.4)	388905 (56.9)	200 (109, 331)	223 (135, 352)	172 (89, 286)	191 (91, 347)
	2019	813643 (21.9)	546266 (67.1)	105 (54, 213)	151 (76, 302)	79 (39, 158)	87 (48, 158)
	2020	637793 (17.2)	481074 (75.4)	82 (43, 161)	128 (67, 226)	68 (39, 123)	55 (32, 93)
	2021	740734 (20.0)	586451 (79.2)	65 (34, 157)	124 (61, 254)	54 (33, 93)	35 (21, 63)
	2022	837307 (22.6)	637373 (76.1)	91 (51, 149)	98 (59, 153)	66 (37, 112)	101 (58, 177)
Age (in Years)	Below 15	228045 (6.1)	152307 (66.8)	111 (56, 213)	136 (73, 244)	78 (42, 153)	80 (40, 166)
	15 – 59	2862414 (77.1)	2045350 (71.5)	97 (48, 194)	131 (70, 239)	74 (39, 146)	75 (37, 158)
	60 and above	622035 (16.8)	442377 (71.1)	89 (45, 180)	123 (65, 226)	71 (38, 138)	70 (35, 148)
Gender	Female	1359926 (36.6)	978225 (71.9)	98 (49, 195)	131 (70, 237)	74 (39, 146)	74 (37, 155)
	Male	2349504 (63.3)	1660109 (70.7)	95 (48, 192)	130 (69, 238)	74 (39, 144)	74 (36, 157)
	Transgender	2284 (0.1)	1460 (64.0)	98.5 (53, 190)	122 (69.5, 227.5)	77 (39, 137)	91 (44, 190)
Notifying Sector	Public	2710721 (73.0)	2124808 (78.4)	93 (47, 187)	127 (69, 233)	71 (38, 140)	72 (36, 154)
	Private	1001829 (27.0)	515260 (51.4)	113 (56, 218)	140 (71, 253)	89 (47, 172)	84 (41, 171)
Site of Disease	Extra Pulmonary	825605 (23.2)	628239 (76.1)	96 (49, 188)	132 (71, 234)	75 (41, 142)	72 (37, 147)
	Pulmonary	2726671 (76.8)	2001515 (73.4)	96 (48, 194)	130 (69, 238)	74 (39, 146)	75 (36, 160)
Drug Type	DSTB	3610266 (97.2)	2559307 (70.9)	96 (48, 191)	129 (69, 236)	73 (39, 144)	73 (36, 155)
	DRTB	102285 (2.8)	80762 (79.0)	127 (65, 262)	168 (88, 318)	94 (51, 184)	107 (53, 228)
HIV status	Non-Reactive	3089052 (83.2)	2382703 (77.1)	91 (46, 182)	123 (67, 226)	70 (38, 135)	72 (36, 150)
	Reactive	65771 (1.8)	44479 (67.6)	92 (48, 187)	146 (76, 258)	76 (42, 140)	89 (43, 186)
	Don’t Know / Missing	557728 (15.0)	212887 (38.2)	180 (93, 313)	196 (106, 332)	147 (73, 264)	167 (79, 316)
Diabetic	Yes	217879 (5.9)	184561 (84.7)	70 (38, 134)	116 (63, 214)	59 (35, 107)	60 (31, 121)
	No	2602310 (70.1)	2044964 (78.6)	91 (46, 179)	122 (66, 222)	70 (38, 130)	71 (36, 148)
	Don’t know / Missing	892362 (24.0)	410544 (46.0)	159 (77, 288)	176 (92, 307)	124 (55, 244)	163 (77, 313.5)
Treatment Outcome	Unfavourable	583173 (16.1)	239041 (41.0)	94 (47, 194)	129 (67, 243)	70 (38, 136)	73 (35, 159)
	Favourable	3032141 (83.9)	2338971 (77.1)	97 (48, 195)	132 (70, 240)	74 (39, 147)	74 (36, 158)

NPY - *Ni-Kshay Poshan Yojana*; TB-Tuberculosis; IQR – Interquantile range; DRTB- Drug resistant TB; DSTB-Drug sensitive TB; HIV – Human Immunodeficiency Virus

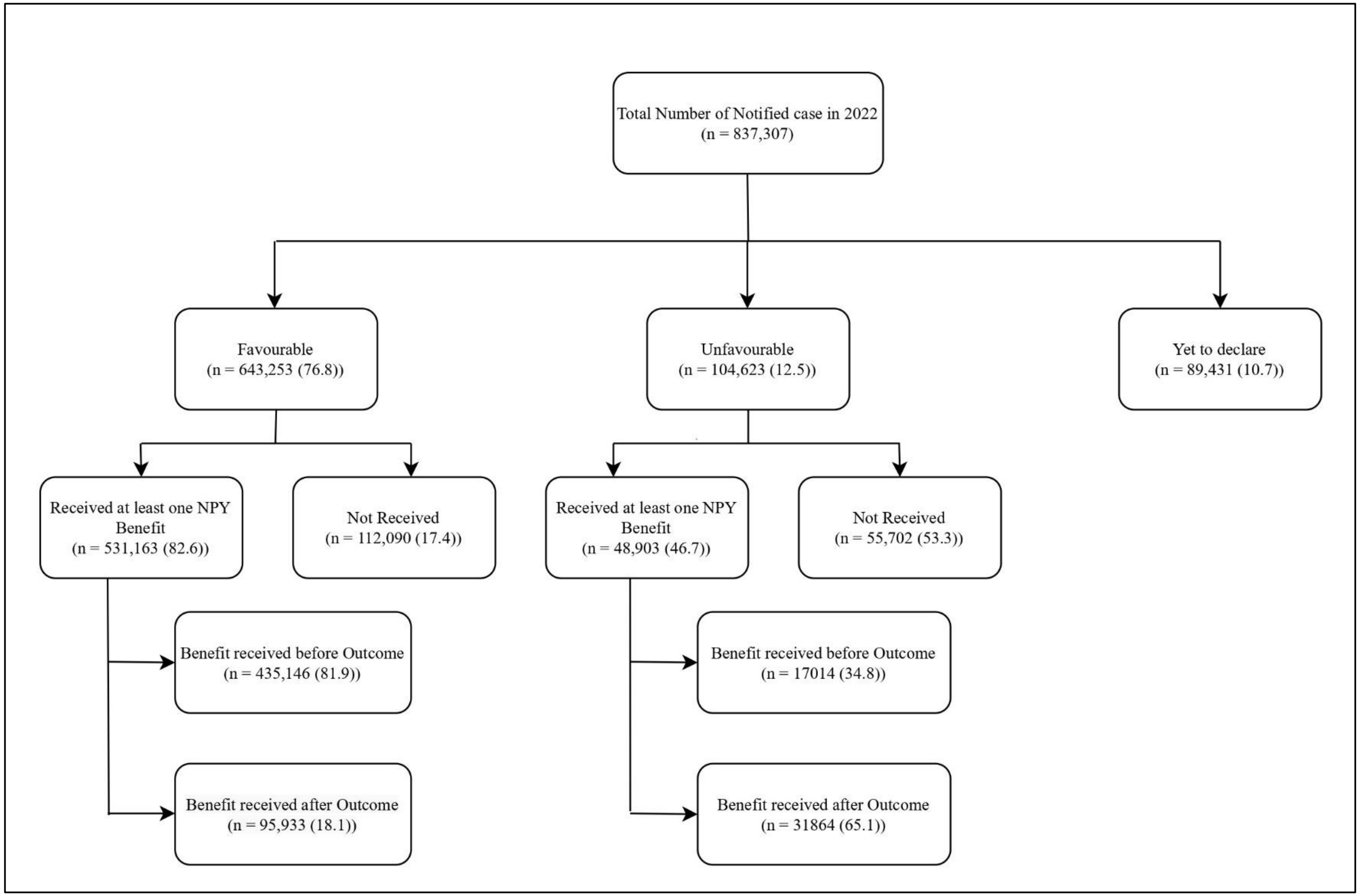

Figure S1 Flow chart describing proportion of patients with TB who had received at least one *Ni-Kshay Poshan Yojana* instalment by treatment outcome, 2022 (N=837307)
Abbreviation: NPY, *Ni-Kshay Poshan Yojana*

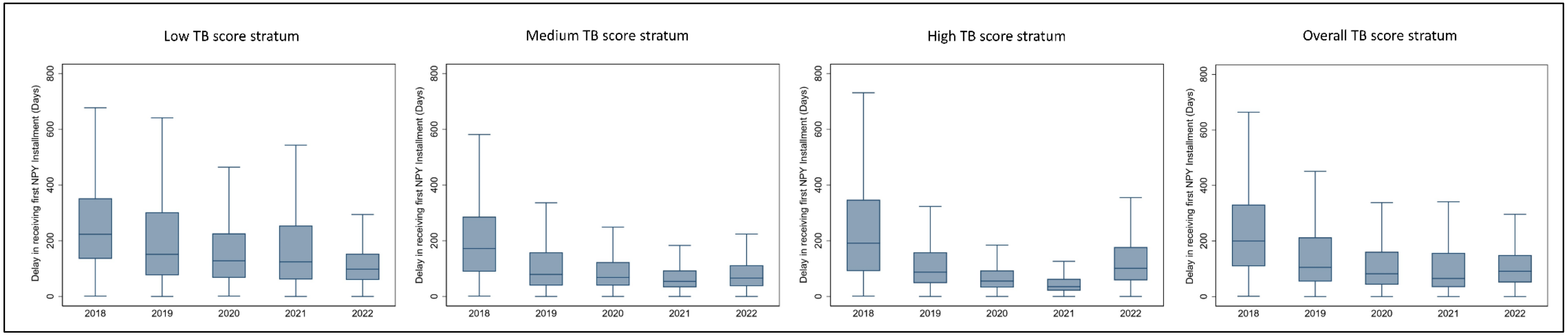

Figure S2 Stratum wise time to receipt of first *Ni-Kshay Poshan Yojana* instalment among patients with TB in India, 2018-2022 (N =3712551)
